# Motivation Measures in Sport: A Critical Review and Bibliometric Analysis

**DOI:** 10.3389/fpsyg.2017.00348

**Published:** 2017-03-09

**Authors:** Rachel B. Clancy, Matthew P. Herring, Mark J. Campbell

**Affiliations:** ^1^Department of Physical Education and Sport Sciences, University of LimerickLimerick, Ireland; ^2^Health Research Institute, University of LimerickLimerick, Ireland

**Keywords:** critical review, sport motivation, questionnaire, measurement, bibliometric analysis, psychometric

## Abstract

Motivation is widely-researched, in both sport psychology and other fields. As rigorous measurement is essential to understanding this latent construct, a critical appraisal of measurement instruments is needed. Thus, the purpose of this review was to evaluate the six most highly cited motivation measures in sport. Peer-reviewed articles published prior to August 2016 were searched to identify the six most highly cited motivation questionnaires in sport: Sport Motivation Scale (SMS), Intrinsic Motivation Inventory (IMI), Situational Motivational Scale (SIMS), Perceptions of Success Questionnaire (POSQ), Behavioural Regulation in Sport Questionnaire (BRSQ), and Task and Ego Orientation in Sport Questionnaire (TEOSQ). The questionnaires were then evaluated and discussed in four sections: Development, Reliability, Correlates, and Summary. Bibliometric data were also calculated (average weighted impact factor) and assessed (e.g., citations per year) to evaluate the impact of the use of each questionnaire. Despite some variance in their psychometric properties, conceptualization, structure, and utility, the six questionnaires are psychometrically strong instruments for quantifying motivation that are widely supported in the literature. Bibliometric analyses suggested that the IMI ranks first and the SMS ranks sixth according to the average weighted impact factors of their original publications. Consideration of each questionnaire's psychometric strengths/limitations, and conceptualization of motivation in the context of specific research questions should guide researchers in selecting the most appropriate instrument to measure motivation in sport. The average weighted impact factor of each questionnaire is a useful value to consider as well. With these points in mind, recommendations are provided.

## Introduction

Motivation can be defined as the force that energizes and directs behavior (Roberts and Treasure, 2001). Thus, it comprises the perceived reasons for engaging in an activity. There is utility in studying motivation, as it provides a theoretical and practical insight into why one initiates, regulates, sustains, directs and discontinues behavior. Studies in education (e.g., Dweck, 1986; Deci and Ryan, 2016), the workplace (e.g., Ambrose and Kulik, 1999; Gagné and Deci, 2005), health and healthcare (e.g., Carter and Kulbok, 2002; Hardcastle and Hagger, 2016), physical activity and exercise (e.g., Buckworth et al., 2007; Gunnell et al., 2014), among other domains, indicate the widespread scale and importance of motivational research. In the area of sport psychology, there is similar interest in the psychological processes that influence behavior, which extends from academia to the playing field.

Motivation is a construct (or latent variable), rather than an observable entity, which contributes to the difficulty in accurately measuring it (Lavallee et al., 2003). Many early assessments of motivation were behavioral in nature or relied on participants to provide verbal reports as to why they engaged in a particular activity. For example, Lepper and Greene (1975) inferred participants' intrinsic motivation by observing their time on task following an experimental intervention. A comparable though less scientific sport-related example is as follows: an athlete who performs extra repetitions in the gym is often perceived by observers as highly motivated, though no measure of motivation has actually taken place. Clearly, methodologically rigorous measurement is needed to assess, understand, and predict the influence of any psychological construct on human behavior (Clancy et al., 2016). Thus, critical appraisal of the strengths and weaknesses of different measurement approaches is essential for our understanding of motivation, and would enhance researchers and practitioners' awareness of subsequent behavior.

Self-report questionnaires are the most commonly used measurement tools in motivation research, with Mayer et al. (2007) identifying over 75 questionnaires on motivation between 1930 and 2005. Specifically, in sport psychology, there is a plethora of motivation questionnaires (Clancy et al., 2016). Although, previous publications have compared the psychometric properties of two instruments (e.g., Lonsdale et al., 2014) or reviewed questionnaires (e.g., Duda and Whitehead, 1998; Vallerand and Fortier, 1998), there is no contemporary peer-reviewed manuscript that provides a comprehensive evaluation of the most widely used self-report questionnaires of motivation in sport psychology. Bibliometric methods (e.g., Lindahl et al., 2015) add depth to such an evaluation by exploring the cited literature in the field. This review sought to address the aforementioned gap by providing a critical appraisal and bibliometric analysis of such measures, and subsequent guidance regarding their use based on the specific research question.

## Methods

Following ethical approval, six databases were searched in order to identify the most highly cited motivation questionnaires in sport prior to August 2016: Academic Search Complete; Google Scholar; PsycARTICLES; PsycINFO; SPORTDiscus; Web of Science. The search was conducted using the following terms:

(motiv^*^ OR regulat^*^ OR behav^*^) AND sport^*^ AND (questionnaire OR measur^*^ OR instrument OR scale).

Reference lists of the obtained articles were searched by hand. The six most highly cited motivation questionnaires in sport were selected for review and are summarized in Table 1: the Sport Motivation Scale (SMS; Pelletier et al., 1995), the Intrinsic Motivation Inventory (IMI; McAuley et al., 1989), the Situational Motivational Scale (SIMS; Guay et al., 2000), the Perceptions of Success Questionnaire (POSQ; Roberts et al., 1998), the Behavioral Regulation in Sport Questionnaire (BRSQ; Lonsdale et al., 2008), and the Task and Ego Orientation in Sport Questionnaire (TEOSQ; Duda, 1989). In order to critically appraise each instrument, further searches were conducted using the questionnaire name combined with test evaluation-related terms (e.g., reliability, psychometric, factor analysis).

**Table 1:** **Overview of six highly cited motivation measures in sport**.

**Construct**	**Measure**	**Original authors**	Items^*^	**Subscales**	**Responses/item**
Motivation	Sport Motivation Scale	Pelletier et al., 1995	28	7	1–7
	Intrinsic Motivation Inventory	McAuley et al., 1989	16	4	1–7
	Situational Motivation Scale	Guay et al., 2000	16	4	1–7
	Behavioral Regulation in Sport Questionnaire	Lonsdale et al., 2008	24	6	1–7
Goal orientation	Perceptions of Success Questionnaire	Roberts et al., 1998	12	2	1–5
	Task and Ego Orientation in Sport Questionnaire	Duda, 1989	13	2	1–5

* *In cases where there are multiple versions of measure, this is the number of items for the most commonly used version*.

Bibliometric data (Table 2) were obtained using the Cited Reference Search in Web of Science. The total number of citations of each original publication was reported, as well as the subset with an impact factor. Some sources (e.g., book series, conference proceedings) had no impact factor and, therefore, were excluded from further calculations. The average weighted impact factor for the original publication of each questionnaire was calculated as follows: (1) the number of articles (citations) in each journal was multiplied by the journal's 2015 impact factor; (2) this value for all the journals was summed and then divided by the total number of articles (citations). This process resulted in a single number describing the impact of the use of each questionnaire.

**Table 2:** **Bibliometric data for six highly cited motivation measures in sport**.

**Measure**	**Original authors**	Citations^*^	**Citations/year**	**Sources with IF**		
				**Journals**	**Articles**	**Average weighted IF**
SMS	Pelletier et al., 1995	393	19.5	120	365	1.53
IMI	McAuley et al., 1989	506	18.7	175	470	1.89
SIMS	Guay et al., 2000	231	13.6	126	202	1.85
POSQ	Roberts et al., 1998	152	8.4	51	142	1.61
BRSQ	Lonsdale et al., 2008	66	8.3	28	61	1.80
TEOSQ	Duda, 1989	221	8.2	72	202	1.55

* *Data obtained using “Cited Reference Search” of Web of Science (Core Collection); IF: impact factor*.

## Results

In the following sections, each measure will be discussed in order of highest to lowest number of citations per year since the original publication date. Although, the bibliometric data (Table 2) indicate that the SMS is the most highly cited questionnaire under review (19.5 per year), the average weighted impact factor of the journals accounting for those citations is the lowest (~1.53). The IMI (~1.89) and SIMS (~1.85) have the highest average weighted impact factors, but many of the SIMS citations are in non-sport journals (e.g., International Journal of Engineering Education, Computers in Human Behavior). As such, the IMI could be interpreted as the questionnaire with the highest impact. Bibliometric data are provided in full in Supplementary Tables 1–6.

In the current review, each measure is evaluated along four domains: (1) the questionnaires are described in *Development*, which outlines background information, structure, updated versions, scoring, and so forth; (2) reliability is briefly summarized in *Reliability*. Cronbach's alpha values are reported as a measure of internal consistency, with 0.70 being the acceptable cut-off for research purposes (Nunnally, 1978). Where possible, indices of temporal stability and model fit are reported. In line with guidelines from Vincent and Weir (1999), test-retest correlations and intraclass coefficients are interpreted as high (>0.90), moderate (0.80–0.90) or insufficient (≤ 0.80); (3) findings regarding the associations between questionnaire scores and related variables are provided in *Correlates*; and, (4) a synopsis of the aforementioned material is presented in *Summary*.

### Sport motivation scale

#### Development

The Echelle de Motivation dans le Sport is a multidimensional and contextual measure of intrinsic motivation, extrinsic motivation, and amotivation toward sport (Briere et al., 1995). Pelletier et al. (1995) used two studies, the first with university athletes and the second with provincial soccer players, to translate and validate this questionnaire into English and, thereby, produce the SMS. The SMS contains seven subscales that measure three types of intrinsic motivation (to know, to accomplish things, to experience stimulation), three types of regulation for extrinsic motivation (identified, introjected, external), and amotivation. Each subscale contains four items, amounting to 28 items in total. In response to criticisms of the SMS, Mallett et al. (2007b) developed the SMS-6, which comprises six subscales. In this measure, the intrinsic motivation subscales were combined into a single subscale, and items were added for integrated regulation, the most self-determined form of extrinsic motivation that was absent from the SMS (Mallett et al., 2007a,b). However, Pelletier et al. (2007) did not conclude that the SMS-6 was superior to the SMS, or even that a revision of the measure was needed. Although the SMS has had a “significant impact on the measurement, prediction, and understanding of sport motivation” (Pelletier et al., 2013, p. 331), a revised version was later developed, namely the SMS-II, to address some of the limitations of the SMS. The 18-item SMS-II, which contains a mix of SMS items and new items, includes a subscale for integrated regulation, and groups the different types of intrinsic motivation into a single subscale.

Scores from the SMS can be provided in three formats. Firstly, a score can be calculated for each subscale, amounting to seven scores per questionnaire. Secondly, subscales can be grouped into broader motivational categories. For example, identified, introjected, and external regulation can be averaged to give one score for extrinsic motivation. Thirdly, a self-determination (or relative autonomy) index can be calculated by assigning weights to each subscale score according to the subscale's position on the self-determination continuum (see Gillet et al., 2010 for an example). Subscale scores (mean followed by standard deviation in parentheses) for the SMS are provided in Table 3. Although there is not a children's version of the SMS, it has been found to have adequate internal reliability with youth athletes (Rottensteiner et al., 2015).

**Table 3:** **Sample composition and subscale scores for a range of papers using the SMS**.

References^*^	**Sample**	**AM**	**EX**	**IJ**	**ID**	**IM-K**	**IM-A**	**IM-S**
Pelletier et al., 1995	319 male & 274 female Canadian university athletes	M: 6.98 (3.10); F: 6.89 (3.00)	M: 11.56 (3.72); F: 10.82 (3.59)	M: 12.29 (3.70); F: 12.46 (4.04)	M: 12.90 (3.15); F: 13.13 (3.24)	M: 12.42 (3.47); F: 13.05 (3.73)	M: 14.17 (3.30); F: 14.88 (3.40)	M: 14.76 (2.99); F: 14.57 (3.49)
Fortier et al., 1995	399 Canadian junior college athletes	5.89 (5.21)	12.68 (5.86)	20.65 (5.07)	17.34 (4.82)	18.80 (6.34)	21.75 (4.96)	22.91 (4.12)
Cresswell and Eklund, 2005	102 professional New Zealand rugby players “pretournament”	1.93 (0.97)	2.88 (1.14)	2.68 (1.05)	3.78 (1.28)	–	4.50 (1.18)	–
Gillet et al., 2010	101 French judokas	1.58 (0.93)	3.06 (1.39)	5.25 (1.19)	4.32 (1.11)	5.19 (0.86)		
Quested and Duda, 2011	392 British dance students	2.69 (1.46)	3.07 (1.27)	3.99 (1.25)	3.36 (1.19)	5.31 (1.00)	5.31 (1.07)	5.67 (0.97)
Rottensteiner et al., 2015	1517 Finnish “persistent” youth athletes	–	2.95 (0.85)	3.34 (0.84)	3.30 (0.85)	3.50 (0.71)		

* *Original SMS; AM, amotivation; EX, external regulation; IJ, introjected regulation; ID, identified regulation; IM-K/A/S, intrinsic motivation-to know/accomplish things/experience stimulation; M, male; F, female; values are mean (standard deviation)*.

#### Reliability

Acceptable internal consistency has been found in most studies using the SMS. Pelletier et al. (1995) reported Cronbach's alpha values of 0.74–0.80, except for the identified regulation subscale (α = 0.63). Cronbach's alpha values of 0.73–0.90 were reported for Canadian athletes (Fortier et al., 1995), 0.72–0.83 for professional rugby players (Cresswell and Eklund, 2005), 0.71–0.85 for French judokas (Gillet et al., 2010), and 0.65–0.87 for British dancers (Quested and Duda, 2011). Mallett et al. (2007a) reported Cronbach's alpha values of 0.78–0.86 for the SMS-6, except for the identified regulation subscale (α = 0.70). Pelletier et al. (2013) reported Cronbach's alpha values of 0.70–0.88 for the SMS-II. Test-retest correlations for the SMS range from 0.58 to 0.84 (Pelletier et al., 1995), which are insufficient to moderate. Confirmatory factor analysis was performed to evaluate the seven-factor structure of the SMS (Pelletier et al., 1995). Although the chi-square statistic suggests a lack of model fit, other statistics (chi-square/degrees of freedom ratio = 1.94; goodness of fit index = 0.94; the adjusted goodness of fit index = 0.92; root mean square residual = 0.048; normed fit index = 0.92) indicate that the model is acceptable (Pelletier et al., 1995).

#### Correlates

Multiple types of correlational data support interpreting scores from the SMS as measures of intrinsic motivation, extrinsic motivation, and amotivation. In line with theoretical predictions, the SMS subscale scores correlate with numerous motivational determinants and consequences. For example, amotivation is negatively associated with perceived competence (determinant), and effort (consequence; Pelletier et al., 1995). Intrinsic motivation is positively correlated with a coach who provides competence-based feedback, and negatively correlated with distraction (Pelletier et al., 1995). Autonomy-supportive coaching positively predicts intrinsic motivation (Pelletier et al., 1995; Gillet et al., 2010; Quested and Duda, 2011). Competitive athletes demonstrate less intrinsic motivation than recreational athletes, reinforcing earlier findings that competition undermines intrinsic motivation due to its emphasis on external rewards (Fortier et al., 1995). Intrinsic motivation is significantly negatively associated with key characteristics of burnout, such as sport devaluation and exhaustion (Cresswell and Eklund, 2005). Contextual self-determined motivation is significantly correlated with situational self-determined motivation (Gillet et al., 2010). Extrinsic regulation positively predicts social physique anxiety among dancers, and amotivation negatively predicts self-esteem (Quested and Duda, 2011). Perceived competence is related to autonomous motivation, which positively influences persistence in team sport (Rottensteiner et al., 2015).

#### Summary

The available evidence supports using the SMS as a measure of intrinsic motivation, extrinsic motivation, and amotivation in sport. A limitation of the SMS is that it does not assess integrated regulation, though this can be overcome by using the SMS-II. The internal consistency of the identified regulation subscale (α = 0.63) is also below the acceptable threshold (Nunnally, 1978). Overall, the SMS is a well-supported, multidimensional questionnaire that is psychometrically sound, brief, and widely used in sport settings.

### Intrinsic motivation inventory

#### Development

The IMI is a multidimensional and situational measure of intrinsic motivation that was first developed for laboratory tasks (Ryan, 1982) and then adapted to sport (McAuley et al., 1989). Thus, it was originally a non-sport questionnaire that McAuley et al. (1989) successfully applied in a competitive sport setting using a sample of university physical education students. In its entirety, it contains 45 items across seven subscales: interest/enjoyment, perceived competence, effort, value/usefulness, felt pressure/tension, perceived choice, and relatedness. A smaller number of IMI items can be selected and modified depending on the activity and research question, without adversely affecting the psychometric properties of the measure. In developing the sport version of the IMI, McAuley et al. (1989) compared two versions containing 16/18 items across four subscales: interest/enjoyment, perceived competence, effort/importance, and pressure/tension. The interest/enjoyment subscale is considered the self-report measure of intrinsic motivation. In contrast, the remaining three subscales account for antecedents (competence) or outcomes (effort/importance, pressure tension) of intrinsic motivation, rather than intrinsic motivation itself. The perceived choice subscale is a common addition to the 16-item version (e.g., Amorose and Horn, 2001). Due to the flexible nature of the IMI, any number of subscale scores can be reported depending on the variable of interest. Accordingly, Table 4 shows scores for each subscale (mean followed by standard deviation in parentheses), and indicates that studies often use a smaller selection of subscales, rather than the maximum number of seven (number of items used is indicated for each subscale). There is not a children's version of the IMI but it has been found to have adequate internal reliability with youth samples (Williams and Gill, 1995).

**Table 4:** **Sample composition and subscale scores for a range of papers using the IMI**.

**References**	**Sample**	**Interest/enjoyment**	**Perceived competence**	**Effort/importance**	**Pressure/tension**	**Perceived choice**
McAuley et al., 1989^*^	116 US college PE students	4.77 (1.44) (5 items)	4.37(1.71) (5 items)	4.47 (1.44) (4 items)	3.04 (1.46) (4 items)	–
Williams and Gill, 1995	174 US middle school PE students	M: 6.17 (0.86); F: 5.83 (0.90) (5 items)	–	–	–	–
Amorose and Horn, 2000^**^	386 US college athletes	6.0 (0.97) (4 items)	5.7 (0.79) (4 items)	6.3 (0.81) (4 items)	4.7 (1.06) (4 items)	5.7 (1.09) (4 items)
Amorose and Horn, 2001^**^	72 US college athletes “preseason”	5.95 (1.05) (4 items)	5.65 (0.95) (4 items)	6.37 (0.84) (4 items)	5.03 (1.45) (4 items)	5.89 (1.11) (4 items)
Reinboth and Duda, 2006	128 British university athletes	–	5.23 (0.77) (5 items)	–	–	–
Pope and Wilson, 2012	102 Canadian university rugby players	–	5.28 (0.90) (5 items)	5.60 (1.20) (4 items)	–	–

* 18-item version (additional item each for interest/enjoyment and perceived competence);

** *20-item version (perceived choice subscale added); M, male; F, female; values are mean (standard deviation); number of items per subscale is included in parentheses*.

#### Reliability

In assessing the suitability of the IMI for use in the sport domain, McAuley et al. (1989) reported Cronbach's alpha values of 0.78–0.84 for three of the subscales, and 0.68 for pressure-tension. The alpha coefficient for the entire measure is 0.85 (McAuley et al., 1989), which is acceptable. Cronbach's alpha values of 0.73 (interest/enjoyment) were reported for American physical education students (Williams and Gill, 1995), 0.62–0.85 for American college athletes (Amorose and Horn, 2000), 0.78 (perceived competence) for British university athletes (Reinboth and Duda, 2006), and 0.85/0.90 (perceived competence/effort/importance) for Canadian rugby players (Pope and Wilson, 2012). For each subscale, the main effect for time across a competitive season is non-significant, demonstrating temporal stability (Amorose and Horn, 2001). The five-factor model of the 16-item IMI was examined using confirmatory factor analysis, and the goodness of fit index (0.788) and coefficient delta (0.76) indicate acceptable fit (McAuley et al., 1989).

#### Correlates

Multiple types of correlational data support interpreting scores from the IMI as measures of different types of intrinsic motivation. Intrinsic interest/enjoyment has a significant positive association with task orientation and perceived competence, and a negative association with ego orientation (Williams and Gill, 1995). Task oriented individuals feel more competent, which leads to greater intrinsic interest and higher effort (Williams and Gill, 1995). Scholarship athletes exhibit greater intrinsic motivation than non-scholarship athletes (Amorose and Horn, 2000). Specifically, scholarship athletes scored higher on perceived competence than non-scholarship athletes, suggesting that being awarded a scholarship enhances intrinsic motivation by reinforcing perceptions of competence (Amorose and Horn, 2000). The influence of coaching style/climate on an athlete's need to feel competent, and the subsequent effects on motivation and effort are well-documented (Reinboth and Duda, 2006; Pope and Wilson, 2012). Similarly, Amorose and Horn (2001) found support for the relationship between perceived coaching behaviors and athlete intrinsic motivation.

#### Summary

The available evidence indicates that scores from the IMI can be interpreted as measures of situational intrinsic motivation in sport. Limitations of the IMI are that it predominantly assesses determinants and consequences of intrinsic motivation, rather than intrinsic motivation itself, and there are no subscales for extrinsic motivation or amotivation. Additionally, the internal consistency of the pressure-tension subscale (0.68) is below the acceptable value of 0.70 (Nunnally, 1978). Overall, the IMI is a very flexible instrument that affords the researcher the opportunity to select/modify relevant items to assess intrinsic motivation in any sport setting.

### Situational motivational scale

#### Development

The SIMS is a multidimensional and situational measure of intrinsic motivation, extrinsic motivation, and amotivation (Guay et al., 2000). It is a state measure (meaning it captures ongoing motivational regulations), focuses on the reasons why people engage in an activity (rather than consequences), and is worded such that it can be used in most settings. It is, however, not specifically a sport questionnaire, meaning it is cited across diverse domains (see Supplementary Table 3). Guay et al. (2000) conducted five studies with university student samples to develop and validate the SIMS, though one of these samples comprised student-athletes. The 16-item scale assesses extrinsic motivation multidimensionally (external and identified regulations), and intrinsic motivation and amotivation as unidimensional constructs. There is also a 14-item version, which may more soundly measure state motivational regulations (Standage et al., 2003). Four subscale scores are generally reported in the literature when the SIMS is used (Table 5; mean followed by standard deviation in parentheses). Although there is not a children's version of the SIMS, it is commonly and successfully used with youth samples (e.g., Podlog et al., 2015).

**Table 5:** **Sample composition and subscale scores for a range of papers using the SIMS**.

**References**	**Sample**	**AM**	**EX**	**ID**	**IM**
Guay et al., 2000^*^	40 Canadian male college students	T: 2.21: R: 2.39	T: 2.65; R: 2.66	T: 3.98: R: 3.34	T: 4.86; R: 4.03
Standage and Treasure, 2002	318 US middle school students	3.91(1.81)	4.88 (1.70)	4.85 (1.59)	4.75 (1.68)
Conroy et al., 2006^**^	165 US youth swimmers	1.72 (1.09)	2.17 (1.43)	5.44 (1.32)	5.80 (1.25)
Gillet et al., 2010	101 French judokas	1.75 (0.96)	3.64 (1.29)	5.09 (1.10)	5.06 (1.08)
Fernandez-Rio et al., 2014	19 Spanish (inter)national swimmers	1.88 (0.90)	2.90 (1.36)	5.67 (1.16)	4.78 (1.35)
Podlog et al., 2015	192 Swedish elite junior skiers	1.87 (1.06)	1.98 (1.04)	6.01 (0.99)	6.25 (0.83)

* Involved experiment with two conditions (task-focused and reward-focused);

** *14-item version (one item dropped each for identified regulation and external regulation); AM, amotivation; EX, external regulation; ID, identified regulation; IM, intrinsic motivation; T, task-focused; R, reward-focused; values are mean (standard deviation)*.

#### Reliability

Internal consistency is largely acceptable for the SIMS. Cronbach's alpha values of 0.62–0.95 and 0.67–0.93 were reported across four studies of college students and one study of collegiate athletes, respectively (Guay et al., 2000). Cronbach's alpha values of 0.83–0.90 were reported for American middle school students (Standage and Treasure, 2002), 0.69–0.90 for American youth swimmers (Conroy et al., 2006), 0.73–0.85 for French judokas (Gillet et al., 2010), 0.80–0.82 for Spanish swimmers (Fernandez-Rio et al., 2014), and 0.63–0.79 for Swedish junior skiers (Podlog et al., 2015). The SIMS has acceptable test-retest reliability, though changes in subscale scores are expected because it is a state measure (Guay et al., 2000). Confirmatory factor analysis of the four-factor structure was performed (Guay et al., 2000), producing a significant chi-square statistic and a non-normed fit index somewhat lower than the 0.90 cut-off value (Bentler, 1995). However, the comparative fit index (0.90) indicates satisfactory model fit (Guay et al., 2000).

#### Correlates

Multiple types of correlational data support interpreting scores from the SIMS as measures of intrinsic motivation, extrinsic motivation, and amotivation at the situational level. In terms of motivational determinants and consequences, intrinsic motivation and identified regulation are positively associated with perceived competence and autonomy (determinants), and concentration, emotions, task interest and behavioral intentions of future persistence (consequences; Guay et al., 2000). The opposite patterns hold true for external regulation and amotivation. Individuals in task-focused experimental conditions report higher intrinsic motivation that those in controlling/reward conditions (Guay et al., 2000). Similarly, a task/mastery orientation is positively associated with self-determined motivational profiles (Standage and Treasure, 2002; Fernandez-Rio et al., 2014). Coach achievement goals affect athletes' achievement goals, which in turn influence their situational motivation (Conroy et al., 2006). Contextual self-determined motivation also impacts situational self-determined motivation and subsequent competitive performance (Gillet et al., 2010). Self-determined situational motivation serves as a mediator between basic psychological needs and athlete engagement (Podlog et al., 2015).

#### Summary

The available evidence supports using the SIMS as a measure of situational intrinsic motivation, extrinsic motivation, and amotivation in sport. A limitation of the SIMS is that intrinsic motivation is assessed unidimensionally, and two types of extrinsic regulation are absent. Overall, the SIMS is a brief, non-sport-specific measure of multidimensional and situational motivation, which can be applied to sport settings due to its open wording.

### Perceptions of success questionnaire

#### Development

The POSQ is a measure of achievement goals in sport that was first formulated using a sample of sport-playing university students (Roberts et al., 1998). In its development, an initial pool of 48 items was reduced to 26, and this version was found to have strong psychometric properties (Roberts et al., 1995). In seeking a more parsimonious scale, Roberts et al. (1998) tested a 16-item version, which was then reduced to 12 items equally divided across two subscales: task and ego orientations. The correlations between the short form and the long form are 0.98 (task) and 0.97 (ego), reinforcing the efficacy of the 12-item version to measure achievement goals in sport. Subscale scores (mean followed by standard deviation in parentheses) for task and ego orientations are provided in Table 6. Though the POSQ has demonstrated adequate reliability among youth samples (e.g., Harwood et al., 2004), there is also a children's version (Lemyre et al., 2002). The original publication found both the adult and children's versions to be reliable and valid instruments (Roberts et al., 1998).

**Table 6:** **Sample composition and subscale scores for a range of papers using the POSQ**.

**References**	**Sample**	**Task orientation**	**Ego orientation**
Ommundsen et al., 1998	148 Norwegian university PE/sport students	4.69 (0.47)	2.87 (0.87)
Pensgaard and Roberts, 2000	69 Norwegian Olympic athletes	M: 4.54 (0.46); F: 4.46 (0.46)	M: 3.95 (0.71); F: 3.90 (0.56)
Lemyre et al., 2002^*^	511 Norwegian male youth soccer players	4.46 (0.52)	3.73 (0.80)
Harwood et al., 2004	573 British elite youth athletes	4.53 (0.49)	3.60 (0.90)
Lemyre et al., 2008	141 Norwegian elite winter sport athletes	4.47 (0.66)	3.86 (0.82)
Rottensteiner et al., 2015	1517 Finnish “persistent” youth athletes	4.17 (0.61)	3.38 (0.88)

* *Youth version; M, male; F, female; values are mean (standard deviation)*.

#### Reliability

Internal consistency of the POSQ is acceptable. For task and ego orientations, respectively, Cronbach's alpha was reported as 0.82 and 0.87 for American university students (Roberts et al., 1998), 0.81 and 0.79 for Norwegian university physical education students (Ommundsen et al., 1998), 0.76 and 0.75 for Norwegian Olympians (Pensgaard and Roberts, 2000), 0.75 and 0.81 for Norwegian youth soccer players (Lemyre et al., 2002), 0.87 and 0.81 for British elite youth athletes (Harwood et al., 2004), and 0.83 and 0.91 for Finnish youth athletes (Rottensteiner et al., 2015). Test-retest reliability is moderate (0.80 for task and 0.78 for ego) across 1 week (Roberts et al., 1998). The two-factor structure was tested using confirmatory factor analysis (Roberts et al., 1998). Despite a significant chi-square statistic, the root mean square residual (0.09) and Tucker-Lewis index (0.90) indicate adequate model fit for the POSQ (Roberts et al., 1998).

#### Correlates

Multiple types of correlational data support interpreting scores from the POSQ as measures of task and ego orientations in sport. Dispositional achievement goals are influenced by the motivational climate, and are related to a host of other variables, such as the perceived purposes of sport, perceived ability, perfectionism, and burnout (Ommundsen et al., 1998; Lemyre et al., 2008). Situational factors (e.g., motivational climate) influence the sources and levels of distress that athletes experience significantly more than dispositional factors (e.g., goal orientations; Pensgaard and Roberts, 2000). Achievement goal orientations affect athletes' sportspersonship attitudes, with a task orientation having a positive effect on moral functioning and an ego orientation decreasing some aspects of sportspersonship (Lemyre et al., 2002). Perceived ability moderates the relationship between goal orientation and sportspersonship, particularly for ego-oriented athletes (Lemyre et al., 2002). Task orientation is important for acquiring and using psychological skills (e.g., goal setting, imagery), though ego orientation can sometimes be adaptive, in that such athletes often engage in useful strategies to pursue their goals (Harwood et al., 2004). Maladaptive motivational profiles, of which an ego orientation is a component, are associated with higher levels of burnout (Lemyre et al., 2008). Goal orientations influence autonomous motivation both directly and indirectly (through their effects on perceived competence), and autonomous motivation then affects persistence in sport (Rottensteiner et al., 2015).

#### Summary

The available evidence indicates that scores from the POSQ can be interpreted as measures of achievement goals in sport. A potential limitation of the POSQ is its use of a five-point Likert scale, which may afford less sensitivity than a seven-point Likert scale. However, each questionnaire in the review could perhaps be improved if it had an even number of response options and was, therefore, forced-choice. With an odd number of response options, respondents can provide neutral data, which can be uninformative. Overall, the POSQ has strong psychometric properties and is easy to administer due to its brevity.

### Behavioral regulation in sport questionnaire

#### Development

The BRSQ is a contextual measure of competitive sport participants' intrinsic motivation, extrinsic motivation, and amotivation (Lonsdale et al., 2008). The measure was developed as an alternative to the SMS, which Lonsdale et al. (2008) found to have somewhat questionable psychometric properties. Four studies were used in questionnaire development, all of which had athlete samples; specifically, the participants in studies one and two were elite athletes (Lonsdale et al., 2008). Rather than modifying existing items from the SMS (as was the case with the SMS-6 and SMS-II), the BRSQ was created from an entirely new pool of items. In total, it comprises 36 items across nine subscales: four subscales for intrinsic motivation (general, to know, to experience stimulation, to accomplish), four subscales for extrinsically motivated regulations (integrated, identified, introjected, external), and a subscale for amotivation. Two alternative versions were compared by the original authors: the BRSQ-8 contains 32 items across eight subscales, and excludes the unidimensional conceptualisation of intrinsic motivation (intrinsic motivation general); the BRSQ-6 contains 24 items across six subscales, and excludes the tripartite conceptualisation of intrinsic motivation (to know, to experience stimulation, to accomplish). In this way, the BRSQ accounts for the multidimensional and unidimensional conceptualizations of intrinsic motivation, as there are subscales for intrinsic motivation and its three corresponding types. A single global score is usually reported for intrinsic motivation in the literature, whereas extrinsic motivation is broken down into its four components (Table 7; mean followed by standard deviation in parentheses). The children's version of the questionnaire is valid among youth athletes (Viladrich et al., 2013).

**Table 7:** **Sample composition and subscale scores for a range of papers using the BRSQ**.

**References**	**Sample**	**AM**	**EX**	**IJ**	**ID**	**IG**	**IM**
Lonsdale et al., 2008^*^	343 New Zealand athletes	2.33 (1.36)	2.03 (1.21)	2.71 (1.60)	5.52 (1.10)	5.55 (1.06)	6.14 (0.92)
Assor et al., 2009^**^	192/9 Belgian students from top sport schools	1.85 (0.88)	2.06 (0.82)	App: 3.55 (0.94); Av: 2.37 (1.01)	4.07 (0.61)	4.27 (0.58)	4.43 (0.54)
Lonsdale and Hodge, 2011^*^	181 New Zealand athletes	2.26 (1.28)	1.91 (1.12)	2.61 (1.46)	5.71 (1.07)	5.55 (1.14)	6.13 (1.00)
Holmberg and Sheridan, 2013^*^	598 US college athletes	2.47 (1.45)	2.59 (1.49)	3.35 (1.72)	5.75 (1.15)	5.50 (1.18)	6.01 (1.14)
Viladrich et al., 2013^***^	7,769 European youth soccer players	1.75 (1.16)	1.89 (1.23)	2.58 (1.45)	4.10 (1.03)	–	4.48 (0.88)
Hancox et al., 2015^*^	1212 UK dancers	2.09 (1.55)	1.80 (1.35)	2.58 (1.86)	5.38 (1.49)	5.46 (1.47)	6.38 (0.92)

* BRSQ-6 (24 items);

** 28-item version (items added for approach/avoidance introjected regulation);

*** *20-item version (no integrated regulation); AM, amotivation; EX, external regulation; IJ, introjected regulation; ID, identified regulation; IG, integrated regulation; IM, intrinsic motivation; App, approach; Av, avoidance; values are mean (standard deviation)*.

#### Reliability

Internal consistency is acceptable for the nine BRSQ subscales, as Lonsdale et al. (2008) reported Cronbach's alpha values of 0.71–0.93 across three studies. Cronbach's alpha was reported as 0.73–0.87 for Belgian students from top sport schools (Assor et al., 2009), and 0.70–0.93 for Canadian athletes (Lonsdale et al., 2009). Test-retest reliability of the subscale scores is supported across a 1-week period, with intraclass coefficients ranging from 0.73 for intrinsic motivation general to 0.90 for integrated regulation and intrinsic motivation to experience stimulation (Lonsdale et al., 2008). Confirmatory factor analysis of the six-factor structure was conducted, producing fit statistics that are generally strong (Lonsdale et al., 2008), and meet cut-off criteria (root mean square error of approximation ≤ 0.06; comparative fit index ≥0.95; Tucker-Lewis index ≥0.95) suggested by Hu and Bentler (1999). Acceptable model fit has also been demonstrated among dancers (Hancox et al., 2015).

#### Correlates

Multiple types of correlational data support interpreting scores from the BRSQ as measures of intrinsic motivation, extrinsic motivation, and amotivation. Autonomous subscale scores are positively correlated with dispositional flow (Lonsdale et al., 2008), and negatively correlated with burnout (Lonsdale and Hodge, 2011; Holmberg and Sheridan, 2013). Burnout may also precede reductions in self-determined extrinsic motivation (Lonsdale and Hodge, 2011). Identified regulation is associated with a more positive pattern of affective and performance correlates (e.g., positive affect, vitality, interindividual performance, intraindividual progress) than introjected regulation (Assor et al., 2009). Furthermore, when valence is considered, introjected avoidance motivation is related to a more negative pattern of correlates than introjected approach motivation (Assor et al., 2009).

#### Summary

The available evidence supports using the BRSQ as a measure of intrinsic motivation, extrinsic motivation, and amotivation in sport. A potential limitation of the BRSQ is that it was designed for use among competitive athletes, thus making it unsuitable for exercise or physical activity settings. This specificity, however, could also be interpreted as a strength. Overall, the BRSQ is an accurate and flexible instrument that facilitates unidimensional and multidimensional measurement through its various versions/subscales.

### Task and ego orientation in sport questionnaire

#### Development

The TEOSQ is an adaptation of an inventory created for scholastic settings (Nicholls, 1989) that assesses individual differences in the proneness for task and ego involvement in sport (Duda, 1989). One study comprising a sample of high school students was used in this process (Duda, 1989). Using 13 items across two subscales, the TEOSQ assesses personal dispositions that are relatively stable (but not fixed) over time. Task scores are typically higher and more stable than ego scores (Duda and Whitehead, 1998). Subscale scores (mean followed by standard deviation in parentheses) for the TEOSQ are provided in Table 8, and readers are directed to Duda and Whitehead (1998) for a table of subscale scores for papers between 1989 and 1997. The TEOSQ has been successfully used for both youth (e.g., Williams and Gill, 1995) and adult (e.g., Lameiras et al., 2014) samples.

**Table 8:** **Sample composition and subscale scores for a range of papers using the TEOSQ**.

**References**	**Sample**	**Task orientation**	**Ego orientation**
Duda, 1989	128 male & 193 female US high school athletes	M: 4.28 (0.47); F: 4.45 (0.80)	M: 2.89 (0.87); F: 2.59 (0.96)
Williams and Gill, 1995	174 US middle school PE students	M: 4.33 (0.57); F: 4.28 (0.54)	M: 2.72 (0.99); F: 2.74 (0.88)
Van-Yperen and Duda, 1999	75 male Dutch soccer students “preseason”	3.90 (0.64)	3.64 (0.73)
Ntoumanis, 2001	247 British university athletes	4.07 (0.44)	3.13 (0.85)
Lameiras et al., 2014	158 Portuguese male professional athletes	4.15 (0.56)	2.71 (0.93)
Allen et al., 2015	177 Scottish (inter)national athletes	4.25 (0.53)	3.54 (0.77)

*M, male; F, female; values are mean (standard deviation)*.

#### Reliability

The TEOSQ has acceptable internal consistency. For the task and ego subscales, respectively, Cronbach's alpha is 0.82 and 0.89 among high school basketball players (Duda, 1989), 0.84 and 0.86 for American middle school students (Williams and Gill, 1995), 0.88 and 0.82 for elite soccer players (Van-Yperen and Duda, 1999), 0.76 and 0.84 for British university athletes (Ntoumanis, 2001), 0.83 and 0.82 for Portuguese professional athletes (Lameiras et al., 2014), and 0.84 and 0.81 for Scottish athletes (Allen et al., 2015). Test-retest reliability of the subscale scores was reported as 0.58 (task) and 0.67 (ego) across one soccer season, which is insufficient (Van-Yperen and Duda, 1999). Numerous investigations containing confirmatory factor analyses support the two-factor structure of the TEOSQ (Duda and Whitehead, 1998).

#### Correlates

Multiple types of correlational data support interpreting scores from the TEOSQ as measures of task and ego orientations in sport. Task orientation is positively associated with beliefs that sport should enhance self-esteem, and encourages effort, mastery, cooperation and rule-following (Duda, 1989). In contract, ego orientation positively predicts views about the extrinsic benefits and personal gains afforded by sport (Duda, 1989), and is positively associated with stronger pro-doping attitudes (Allen et al., 2015). The association between task orientation and cooperation/prosocial behavior was also reported by Lameiras et al. (2014). There are direct and positive links between task orientation, perceived competence, intrinsic motivation/interest and effort (Williams and Gill, 1995). Task orientation is also related to the belief that effort contributes to achievement, whereas ego-oriented athletes believe that ability/talent determines success (Van-Yperen and Duda, 1999). There is a further link between task orientation and season-long performance improvement (Van-Yperen and Duda, 1999). Ntoumanis (2001) found that task orientation predicts motivational variables high in self-determination, whereas ego orientation predicts the opposite.

#### Summary

The available evidence indicates that scores from the TEOSQ can be interpreted as measures of task and ego goal orientation in sport. As with the POSQ, a potential limitation of the TEOSQ is its use of a five-point Likert scale, which offers fewer response options than a seven-point Likert scale. Additionally, the test-retest reliabilities are low. The TEOSQ is a psychometrically sound instrument for measuring dispositional goal orientations that has been used extensively in sport settings without argument for any revisions.

## Discussion

This review set out to evaluate the six most highly cited motivation measures in sport. Each questionnaire attempts to capture the reasons underlying behavior in the sport domain, thereby assessing motivation in a broad sense. However, there is a distinctive difference between how motivation is conceptualized in each questionnaire. The SMS, IMI, SIMS, and BRSQ consider intrinsic motivation, extrinsic motivation, and/or amotivation. In contrast, the POSQ and TEOSQ adopt a goal perspective approach in their measurement of motivation. It is important to note that this distinction does not confer an advantage to one type of questionnaire over another. Rather, it is simply an essential element for the researcher to consider prior to deliberating the relative merits of a particular questionnaire. Should a researcher wish to quantify intrinsic and extrinsic motivation, the SMS, IMI, SIMS, or BRSQ would be suitable. In contrast, a researcher who would like to account for personal goals in their measurement of motivation may priorities the POSQ or TEOSQ. In addition to conceptualization, differences in development, scoring, and youth administration should be deliberated when appraising motivation measures in sport. Bibliometric data can also be useful for indicating the impact of the use of a specific questionnaire, though other methodological features and research design issues must also be considered (Clancy et al., 2016). In the current review, the IMI ranks first and the SMS ranks sixth according to their average weighted impact factors.

In terms of development, there are three distinct groups among the six questionnaires. First, the SIMS stands alone because it is not a sport-specific questionnaire, though it can be adapted for that purpose. Next, the IMI was originally a non-sport questionnaire, but it was modified for the sport domain, which is the version included in this review. Thirdly, the remaining four questionnaires were created specifically for sport. In addition to these distinctions, there are differences in development based on the sample used in the original publications. Five of the publications (SMS, IMI, SIMS, POSQ, TEOSQ) comprised student samples, four at university-level and one at high school-level, which could be indicative of convenience sampling. In contrast, the BRSQ was developed from data from elite and non-elite athletes, which may have been advantageous when developing a measure for this group. Scoring procedures for each questionnaire are straightforward, though the SMS provides more flexibility because subscale scores can be combined to give a single global score, which is frequently reported in the literature and contributes to its ease of use. Thus, while all of the measures provide component scores, only the SMS offers an established method for producing a single score for each participant. As a final comparison, the POSQ and BRSQ have children's versions available, which is ideal when examining youth samples. The remaining four questionnaires, however, have adequate internal reliability when administered to children, indicating their utility among participants of all ages.

The reviewed instruments account for the different conceptualizations of motivation, and are applicable at either the contextual or situational level. Although they vary in their development, scoring, and administration, the conceptualization and level of applicability are likely the most important considerations for researchers selecting a questionnaire. The SMS and BRSQ assess intrinsic motivation, extrinsic motivation and amotivation at the contextual level, and allow for intrinsic motivation to be measured as a unidimensional or multidimensional construct. Next, the IMI and SIMS adopt contrasting approaches to measuring situational motivation. The IMI facilitates an in-depth view of situational intrinsic motivation only, whereas the SIMS quantifies intrinsic motivation (unidimensionally), extrinsic motivation, and amotivation. Lastly, the POSQ and TEOSQ assess task and ego goal orientations in sport. It is clear that researchers have several questionnaires to choose from when attempting to answer a specific research question. Although each of the questionnaires reviewed here has limitations, they predominantly exhibit strong psychometric properties and are widely used, reinforcing their utility for measuring the underlying *why* of behavior in sport.

Valid and reliable measurement is a precursor to the understanding of any psychological construct. Although an unobservable variable can be challenging to measure, the enduring interest of researchers and practitioners in motivation has resulted in the development of numerous instruments for quantifying it. The six questionnaires are psychometrically strong self-report tools for assessing motivation that emerged between the late 1980s and late 2000s, and continue to be widely cited in sport psychology. As previously mentioned, there are clear distinctions between the questionnaires that make them applicable to certain research questions and not others. When considered as a group, however, the IMI has the greatest impact in terms of its use. The SMS ranks sixth in this regard, though it is the most highly cited instrument under review. It is evident that bibliometric analysis enhances the ability to critically appraise questionnaires, and moves understanding beyond simple description. As such, the current review contributes to the field of sport psychology by filling a gap in measurement-related literature, and providing objective guidance for researchers and practitioners who wish to quantify motivation. It may also indicate fruitful avenues for the development of future questionnaires or alternative methods to assess motivation in sport.

## Author contributions

RC, MH, and MC have satisfied all the criteria for authorship: substantially contributing to the reviews conception and interpretation; drafting and revising the work; approving the version to be published; agreeing to be accountable for the work.

## Funding

This work was supported by the Irish Research Council [GOIPG/2015/2665].

### Conflict of interest statement

The authors declare that the research was conducted in the absence of any commercial or financial relationships that could be construed as a potential conflict of interest.
